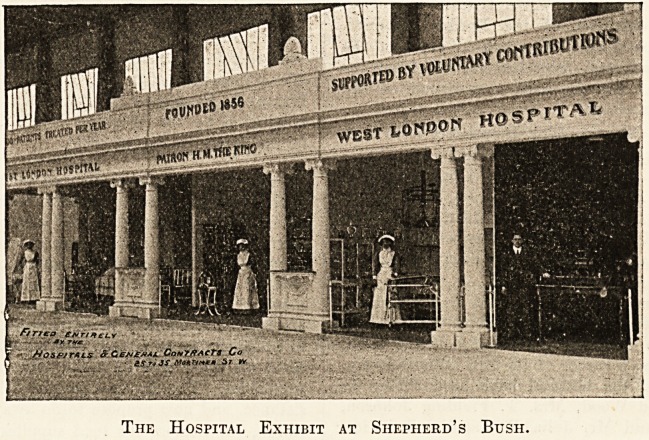# New Appliances and Things Medical

**Published:** 1910-07-09

**Authors:** 


### NEW APPLIANCES AND THINGS MEDICAL.

[We shall be glad to receive at our Office, 28 & 29 Southampton Street, Strand, London, W.C., from the manufacturers, specimens of all new preparations and appliances.]

### A HOSPITAL EXHIBITION AT SHEPHERD'S BUSH.

A somewhat imposing structure, representing the West London Hospital, is situated in the Machinery Hall at the Japan-British Exhibition. It consists of four sections exhibiting the most important departments of a general hospital. Section I. represents the Hospital Ward, completely fitted and equipped with the most modern and up-to-date requirements. The walls are covered in Kent's Crystopal tiling, and the floor is composed of Terrona jointless flooring. All corners of the floor, walls, and ceiling are rounded in accordance with the latest hygienic principles. Here can also be seen the Skeffington Patent Bed-lifter, an excellent device for lifting a bed-ridden patient, which is controlled by the turning of a single handle.

Section II. exhibits the Ward Kitchen where demonstrations on invalid cookery are given daily. This represents a model of all that such a department should be. Here again, the walls are of Crystopal tiling and the flooring used is that of the Terrona, which is employed throughout the building.

Section III. shows the Operating Theatre, furnished entirely on the latest and most modern style in the patent unchippable enamel and glass. A model operating table is on view which possesses many advantages; among these may be mentioned the Teskie patent centre adjustment for raising or lowering the table, which allows surgeons of different stature to work in equal comfort, in fact, almost any position can be easily and rapidly secured, and not the least of its acquisitions are the shoulder supports, which prevent slipping in the Trendelenburg position and render the patient's position more sure and comfortable. An excellent specimen of a high-pressure steriliser is shown, and also sterilisers for instruments, bowls, trays, etc.; these are heated by electricity. The lighting of the theatre is of exceptional power and provided by the Nernst electric lamp with several adjustments which enables the light to be thrown in any desired direction. The sanitary fittings in this theatre are by Messrs. Dent and Hellyer, and the sinks can be worked either by the foot or shoulder action; in fact, the entire fittings of this department are executed in the most up-to-date methods, and special mention should be made of the new sterilising drums which are joined entirely without the aid of solder and arranged on quite a new principle.

Section IV. gives an example of an $x$-ray Department. Here lectures, lasting a quarter of an hour, are given.

The Hospitals and General Contracts Co., of Mortimer Street, W., is responsible for the scientific appliances, instruments, and general furniture, all of which are splendid specimens of hospital requisites, and conclusively prove the excellence of this firm's manufactures and the exceptional ability with which it keeps abreast with the very last improvements in the fittings and general equipment of a modern hospital.

It may be of interest to our readers to know that the Hospitals and General Contracts Co. is at the moment arranging for the manufacture and sale of Dr. Stansfield Collier's patent bedstead.

The Hospital Exhibit at Shepherd's Bush.

### JACKSON'S SELF-FEEDING WATER-BOILER.

(The Jackson Boilers, Ltd., Queen Square, Leeds.)

Hot water is a necessity in every hospital and nursing home, and any apparatus that will enable a small quantity of pure water, just at boiling point, or at any temperature that may be desired, to be obtained at any time, is a boon.

In the Jackson boiler, which is heated by gas, it is claimed that a supply of boiling water can be had at short notice, at any time of the day or night, at small cost. In the apparatus itself, the inventor, Mr. Henry Jackson, has applied the latest scientific principles, those upon which modern steam-boilers are largely built, and those upon which the latest forms of gas-burning apparatus are based. Burners of the ordinary white-flame type are employed, the gas being ignited when required to heat the water, by a pilot light, as with the well-known Welsbach mantle. Mr. Jackson does not believe in automatic gas-cocks turning the gas on and off as the water is drawn, but he has a very simple arrangement, by which the gas is turned on when boiling water is being drawn. The boiler is self-feeding, a specially arranged ball-valve being provided, out of reach of the hot water. The whole of the gas delivered by the burners is usefully consumed, so that there is no smell of unburnt gas coming from it. The heating portion of the boiler is arranged to give a large heating-surface, and to provide baffles, detaining the products of combustion of the gas in such a manner that the whole of the gas is consumed, and the metal in contact with the water is maintained at a high temperature. The boiler is constructed wholly of copper, very carefully and strongly put together, and a special form is made for use where only hard water is available.

According to some tests made by gas engineers for their own information, a gallon of cold water can be raised to boiling point with a consumption of gas of only 4 cubic feet, and after the water has been raised to the boiling temperature any quantity can be obtained at the expense of 2½ cubic feet for every gallon of water boiled. It will be seen from these figures to what a very high state of efficiency the Jackson boiler has been raised. It may be mentioned that the special form designed for use with hard waters is easily and quickly cleaned.

### DOWN BROS. AT THE BRUSSELS EXHIBITION.

The exhibit of British surgical instruments at Brussels compares in the most gratifying manner with anything shown by other nations. It is that of only one firm, that of Down Bros.; but it is sufficiently representative, and attracts a good deal of attention. The evident superiority of the British instruments is found in the high finish and the comparative lightness of the appliances. They admit of more delicate manipulation, a merit of the greatest importance for surgery. It is, perhaps, unlikely that special foreign publicity will be given to this fine British exhibit, since the wider use of any successful implement depends far less upon advertisement, even at home, than upon its being seen in use or recommended by one surgeon to another. International intercourse in the profession does occur; and at the Brussels Exhibition Great Britain is so worthily represented that this section attracts cultured visitors of every sort. The firm shows a choice and large selection of its well-known manufactures in surgical instruments, hospital furniture, and sterilising apparatus. The collection consists exclusively of new ideas, or modifications or improvements of existing models, carried out by the firm for or under the direction of leading British surgeons. The instruments are all London made in the firm's own factory by skilled British artificers, and so may be said to be thoroughly representative. It is an installation that affords an indication of the progress made in recent years by British ingenuity and handicraft in an industry which, for humanity's sake alone, must always call forth the highest efforts, and in which firms of standing recognise a first duty to guard from any deteriorating influence. One of the most interesting exhibits is the "First-Aid" set, as designed for his late Majesty King Edward VII., intended principally for motorists. This useful companion is equally serviceable for accidents at race meetings, in large crowds, or in the home; and clearly worded instructions in heavy type for each compartment explain the use of the contents.

In the furniture section are shown operating tables, manufactured under the firm's patents, with mechanism for rendering them either capable of travelling smoothly on their castors, or firmly fixed at one spot, and for accommodating the height and position of the table and patient to the requirements of the various operations. This section contains also steel and glass cabinets, travelling and fixed electric light standards and pendants, with arrangements for raising, lowering, and varying the position of the light as required; and many other useful contrivances for ward and theatre use.

## Figures and Tables

**Figure f1:**